# Editorial: Extreme environmental microbial products: Structures, functions, biosynthesis

**DOI:** 10.3389/fmicb.2023.1122106

**Published:** 2023-02-02

**Authors:** Junfeng Wang, Xianwen Yang, Li Liao, Martina Cappelletti, Mirko Basen

**Affiliations:** ^1^CAS Key Laboratory of Tropical Marine Bio-Resources and Ecology/Guangdong Key Laboratory of Marine Materia Medica, South China Sea Institute of Oceanology, Chinese Academy of Sciences, Guangzhou, China; ^2^State Key Laboratory Breeding Base of Marine Genetic Resources, Third Institute of Oceanography, State Oceanic Administration, Xiamen, China; ^3^Key Laboratory for Polar Science, Ministry of Natural Resources, Polar Research Institute of China, Shanghai, China; ^4^Department of Pharmacy and Biotechnology, University of Bologna, Bologna, Italy; ^5^Institute of Biosciences, Faculty of Mathematics and Natural Sciences, University of Rostock, Rostock, Germany

**Keywords:** extremophiles, antitumor, anti-inflammatory, pro-angiogenic, functional substances

Microbial products such as penicillin and cyclosporine and others have made phenomenal contributions to the health and wellbeing of people throughout the world. There is growing evidence that microbes can provide bioactive natural products for drug discovery and development, which account for half of all pharmaceuticals on the market. Nevertheless, as research progressed, untapped environmental microorganism resources became increasingly scarce. Chemical, ecological, and pharmacological investigations on microorganisms from extreme environments have increased significantly in recent years. Extreme habitats are characterized by extreme temperatures, ultraviolet (UV) radiation, pH, salt content, or other factors. Faced with multiple extreme environmental stresses, extremophiles have evolved specialized genetic capabilities to produce novel bioactive products. Microbial products from extreme environments have important roles in adaptation to environments, in species communication, and in biotechnological as well as pharmaceutical applications.

Extremophiles continue to attract the attention of chemists, ecologists and pharmacologists, resulting in the stimulation of numerous initiatives to study the structure, synthesis and pharmaceutical applications of products from extreme environments. Under the Research Topic “Extreme environmental microbial products: Structures, functions, biosynthesis,” a total of 11 articles were published, covering a variety of topics involving marine microorganisms including microbial isolation from different habitats and the discovery of novel, bioactive functional substances such as chaetofanixins A–E, adametizine C, streptocarbazoles F–H, and QsGH13 (a alpha-glycosidase). Also, new microbial enzymes were reported, focusing on structure, function, and thermo-halo-alcohol adaptation of enzymes in extreme environments.

Halotolerant and acidotolerant fungi isolated from mangroves have been extensively studied (Cai et al.; Ren et al.; Wang W. et al.; and Wang G. et al.). Cai et al. investigated the antitumor and anti-inflammatory components isolated from the mangrove soil-derived fungi *Penicillium ludwigii* SCSIO 41408. Adametizine C displayed significant inhibitory activities against LPS-induced NF-κB with IC_50_ value of 8.2 μM. Ren et al. evaluated the potential anti-inflammatory candidate, 5-chloro-6-hydroxymellein, which further alleviated the pathological lung injury of lipopolysaccharide (LPS)-administrated mice and protected RAW264.7 macrophages against LPS-induced inflammation through the phosphatidylinositol 3-kinase/protein kinase B (PI3K/AKT) pathway *in vivo*. Also, eschscholin B and daldilene A showed significant cell-based anti-inflammatory activities reported by Wang G. et al.. Further more, eschcholin B suppressed the release of LPS-induced iNOS and COX-2 inflammatory cytokines and the MAPK and NF–κB signaling pathways participated in the regulation of LPS-induced inflammatory processes. Wang W. et al. discovered seven pairs of azaphilones *E*/*Z* isomers from the mangrove-derived fungus *Penicillium sclerotiorum* HY5. Among them, some azaphilone derivatives exhibited potent phytotoxicity against the growth of radicle and plumule on *Amaranthus retroflflexus* L.

In the past 50 years, about 20,000 natural products have been reported from marine microorganisms. However, the number of natural products reported from deep-sea microorganisms is very low, and microorganisms from hadal trench environments (>6,000 m) are seldom investigated. Fan et al. isolated a fungal strain YP-106, identified as *Chaetomium globosum* from the hadal seawater collected at a depth of 6,215 m from Yap Trench in the western Pacific Ocean. Chaetofanixins A-E produced by this fungus were identified as azaphilone derivatives characterized by a pyranoquinone oxabicyclic skeleton. Using the zebrafish screening model, new chaetofanixins A–E showed excellent pro-angiogenic activities in a dose-dependent manner, possessing the potential to be developed as natural cardiovascular disease agents.

Xia et al. identified a fungus *Pestalotiopsis* sp. XWS03F09 isolated from the sponge *Phakellia fusca* collected in Xisha Islands; secondary metabolites from this strain included the ergosterol compound LH-1. Melanoma cells were more sensitive to LH-1 than other cancer cell lines. The IC50 value of LH-1 on B16-F10 melanoma cells was 16.57 μM at 72 h, compared to IC50 values >60 μM at 72 h for other cell lines. Further investigations of biological activities against melanoma *in vitro* and *in vivo* showed that LH-1 could inhibit proliferation and migration of cancer cells, induce apoptosis *via* the mitochondria apoptotic pathway, and upregulate *OBSCN* gene expression in melanoma cells.

Pelagic microorganisms can produce highly active secondary metabolites. Xia et al. identified a fungus *Pestalotiopsis* sp. XWS03F09 isolated from the sponge *P. fusca* collected in Xisha Islands; secondary metabolites from this strain included the ergosterol compound LH-1. Melanoma cells were more sensitive to LH-1 than other cancer cell lines. The IC_50_ value of LH-1 on B16-F10 melanoma cells was 16.57 μM at 72 h, compared to IC_50_ values >60 μM at 72 h for other cell lines. Further investigations of biological activities against melanoma *in vitro* and *in vivo* showed that LH-1 could inhibit proliferation and migration of cancer cells, induce apoptosis *via* the mitochondria apoptotic pathway, and upregulate *OBSCN* gene expression in melanoma cells.

The Yellow mushroom (*Floccularia luteovirens*) grows widely on the Qinghai-Tibet Plateau, a high-altitude habitat characterized by extreme ultraviolet irradiation. Guo et al. investigated the adaptation of *F. luteovirens* to strong UV irradiation, for example the production of the vitamin riboflavin.

Bacteria are important producers of antibiotics and other allopathic active substances. Under the guidance of global natural product social (GNPS) molecular networking, Cui et al. identified a staurosporine-producing strain, *Streptomyces* sp. OUCMDZ-5380, and characterized three new indolocarbazoles as streptocarbazoles F–H. These three new compounds showed a selective antiproliferation on the acute myeloid leukemia cell line MV4–11 with the IC_50_ values of 0.81, 0.55, and 1.88 μM, respectively. Wen et al. isolated the *Bacillus amyloliquefaciens* strain W0101 with remarkable antifungal activity from a sediment sample of the Arctic Ocean. An antifungal peptide (W1) with potential applicability in the biocontrol of plant diseases was purified and shown to be derived from the fragment of preprotein translocase subunit YajC.

Some biological enzymes, derived from special habitats, have some unique physical and chemical functions. Zhai et al. detected a novel GH13 family α-glucosidase, QsGH13, from the deep-sea bacterium *Qipengyuania seohaensis* sp. SW-135. QsGH13 is highly substrate specific and only hydrolyzes sugars containing alpha-1,4 glucoside bonds. Cen et al. reported two loops of exo-inulinase InuAMN8, which was isolated from a cold-adapted bacterium *Arthrobacter* sp. MN8. Inulin is an abundant water-soluble storage polysaccharide after starch in nature. Elucidation of the structure of exo-inulinases is helpful for its application in the hydrolysis of inulin.

## Author contributions

All authors listed have made a substantial, direct, and intellectual contribution to the work and approved it for publication.

